# Scientific misconducts: paper mills in Peru

**DOI:** 10.17843/rpmesp.2022.394.12473

**Published:** 2022-12-22

**Authors:** Percy Mayta-Tristán, Ruben Borja-García

**Affiliations:** 1 General Directorate of Research, Development and Innovation, Universidad Científica del Sur, Lima, Peru. Universidad Científica del Sur General Directorate of Research Development and Innovation Universidad Científica del Sur Lima Peru

Peru has increased its scientific production in recent years as a result of the 2014 university reform and the licensing process of universities and medical schools by the National Superintendence of Higher Education (SUNEDU) ^1^^,^^2^. In addition, institutions with higher scientific production are better positioned in university rankings ^3^^,^^4^; likewise, their teachers and students are recognized as researchers (RENACYT researcher) by the Peruvian National Council for Science, Technology, and Innovation (CONCYTEC) ^5^.

This situation has led to a greater interest among the university community regarding research and publication in indexed journals, with changes in research training strategies such as the acceptance of theses in scientific article format ^6^^,^^7^, development and support for research groups ^8^, as well as creating incentives for teachers who publish ^9^, including the bonus for research teachers that exists by law for public universities ^10^, among others.

One of the consequences of these changes is that both researchers and institutions may favor the publication of certain types of manuscripts to increase their production, such as conferences in congresses (proceeding papers) and letters to the editor (particularly commentary letters, on the same topic, published in different journals), which are more rapidly published, and are reflected in a growing trend in the country, especially the former (Figure 1).

**Figure 1:**
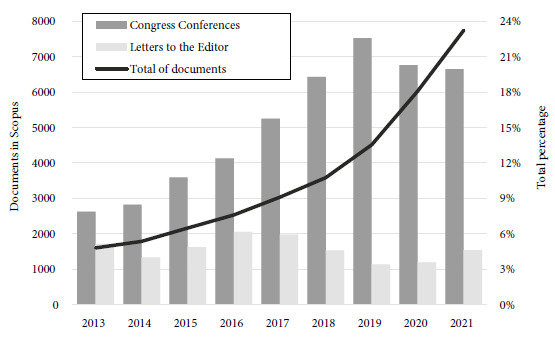
Evolution of the publication of documents by Peruvian authors in Scopus according to type of document, Scopus 2013-2021.

Research incentive and recognition policies have a positive purpose for the institutions, the country, and the researchers. However, there are at least five elements in this Peruvian process that are a potential breeding ground for research malpractices to occur if there are not adequate controls:

1. In 2018, CONCYTEC included in the evaluation criteria for RENACYT researchers, in addition to selective and recognized databases such as Scopus, Core Collection of Web of Science (Science Citation Index Expanded, Social Science Citation Index) and SciELO, a diversity of publishers and databases (e.g., Latindex) that did not have the same filters as the others; CONCYTEC also included research books in the score.
2. A regular professor at a public university who is recognized as a RENACYT researcher by CONCYTEC receives a bonus corresponding to 50% of his or her salary from the Ministry of Education and a reduction of his or her teaching time to one course per year.
3. Universities, mostly private, provide bonuses to their professors and researchers if they publish manuscripts in indexed journals.
4. For the new institutional licensing process by SUNEDU, as well as for human medicine licensing, it is required to accredit a percentage of teachers who are RENACYT.
5. A greater concern of universities to appear and be in a better position in university rankings.

This has contributed to a “publish or perish” culture that can lead to bad scientific practices such as authorship issues (gifted, invited) ^11^, redundant publication ^12^, plagiarism ^13^, data manipulation ^14^, as well as publication in predatory journals ^15^.

At least 1% of the production from the nine universities with the highest scientific output is found in potentially predatory journals indexed in Scopus; there is a growing trend over the years, and it is suspected that this may occur more frequently in other universities with a recent interest in research ^16^. Predatory journals prioritize self-interest, and their sole purpose is profit. They are characterized by presenting misleading or false content, do not follow good editorial and publication practices, lack transparency, and use aggressive strategies to seek articles for publication ^17^. In addition, they tend to have -if they do have it- a quick and insubstantial peer review process that does not guarantee the quality of the content; they also have short publication periods ^(^^18^^,^^19^.

In recent years, along with the sale and purchase of theses ^20^, there has been a proliferation of advertisements from local companies dedicated to “helping” university professors to become RENACYT researchers, offering them, in the best scenario, to publish their studies or theses in journals included in Latindex, often linked to the same company. In other cases, they offer the entire research process up to publication, without the certainty that there was a real study behind it (there would be manipulation of data or plagiarism). Advertisements have also been detected from companies that offer and buy authorships, the cost of which increases as the level of indexing and impact of the journal increases.

These types of fraudulent organizations are referred to as “paper mills” and are a recent and growing global problem, aimed at distorting the scientific research and publication process ^11^^-^^14^. The Committee of Publication Ethics (COPE) describes it as “process by which manufactured manuscripts are submitted to a journal for a fee on behalf of researchers with the purpose of providing an easy publication for them, or to offer authorship for sale” ^21^. These manuscript mills may include one or more of these practices, which are grouped into manuscript ghostwriting, simulation of the research process, buying and selling manuscripts, and manipulation of the research process (Table 1) ^22^.

**Table 1:** Typification of manuscript factory

Practices of manuscript mills (scientific articles)	
1	Manuscript ghostwriting: another person who is not part of the list of “authors” writes the manuscript to be published. It can have different modalities:
	“Pseudo-original” manuscripts that include data that are not their own, which can be false, stolen or manipulated; it also includes the manipulation of images. They may be systematic reviews with or without meta-analysis, bibliometric studies or proceeding papers.
	Review articles, commentaries, essays, or letters to the editor.
	Translation of manuscripts first published in a language other than English and then submitted to a journal in English, or vice versa (particularly in the case of theses, from English to Spanish).
	Theses and academic research papers with or without real data. They use the term “consulting” as a front for ghostwriting. ^b^
2	Simulation of the research process, mainly for thesis or institutional work. ^b^
	Elaboration of the research protocol and management of the approval and support process. ^b^
	Provision of plagiarized or fraudulent databases.
3	Purchase and sale of manuscripts.
	Purchase of articles from researchers, in order to offer the complete article or authorship positions.
	Sale of authorship or authorship position in manuscripts in which they have not participated.
4	Manipulation of the publication process.
	Ensure rapid publication in journals managed by them or related companies, without a review process. In some cases, these journals officially do not charge for publication so as not to be considered predatory. ^b^
	Fake peer review or manipulation of the review process, including the possibility of impersonating reviewers by sending fake email accounts of real researchers when suggesting reviewers for the article.
	Selection, submission, and follow-up services to scientific journals, particularly to predatory journals linked or not linked to them. This may include the use of a fake “corresponding author2 email address or the direct management of an email account that is not that of the “corresponding author”.
	Publication of congress lectures (proceeding papers) managed by themselves. ^b^
	Publication of “research books” which may be a thesis or own or simulated research in the format of a book with ISBN managed by them. May include a fake peer review certificate. ^b^

a Adapted from Perez-Neri *et al*^22^.

b Added from the practices evidenced in Peru.

It is necessary to typify this kind of malpractice, which in turn includes other kinds within itself, in order to detect and sanction it. In the first place, it is necessary to identify the manuscript mills operating in the country and the journals associated with them. Likewise, the people who promote them, who, if they are researchers affiliated to an academic institution, should be investigated, and sanctioned.

In some cases it is possible that the teacher, out of ignorance, acquires this service thinking that it is a form of support for the writing and publication of articles, but in other scenarios there is a direct intention to commit fraud since they seek to be authors of research that they have not done or knowing the processes they prefer this way to quickly reach the goal of being recognized as a researcher. As in the case of plagiarism ^23^, the typification of the “use of manuscript mills” malpractice exists, and the sanction will depend on the aggravating and extenuating factors identified during the investigation process.

Currently, the change in the requirements to be a researcher recognized by CONCYTEC has partially solved the problem by no longer including Latindex in the classification ^5^; however, two challenges remain regarding the control of the editorial function of these manuscript mills. The first aspect is that some of these fraudulent companies have journals that are in Latindex and, since they are no longer an indicator for evaluation by CONCYTEC, they will request their inclusion in SciELO or in the Emerging Source Citation Index of Web of Science, which could be more accessible than entering Scopus or the Science Citation Index Expanded. Secondly, some offer the publication of “research books” and even scientific congresses where they guarantee the publication of the work in their journals or as books or chapters that they also edit (Table 1). CONCYTEC through its National Committee of Scientific Integrity should try to identify, investigate, and sanction them when appropriate.

To discourage the use of manuscript mills, universities should train their faculty and students on the correct processes of research and publication, but it is also necessary to identify, investigate and sanction those who use these services. For this purpose, the Scientific Integrity Offices/Units/Committees should function properly, which are different from the Institutional Research Ethics Committees, since unlike the latter - which seek to protect the research subject - the former watch over good research practices and have the power to investigate and sanction those who commit misconduct ^24^^,^^25^.

Finally, it is necessary to continue with the policies already implemented to promote research, since they contribute to the country’s development. Integrity and honesty are inherent characteristics of research work, both at the level of individuals and institutions. Therefore, bad practices should be identified, investigated, and sanctioned, with emphasis on those that are growing, such as manuscript mills.

## References

[1] Zegarra Rojas O (2019). Modelo de licenciamiento de los programas de pregrado de medicina en el Perú. Acta Med Peru.

[2] Mayta-Tristán P, Toro-Huamanchumo CJ, Alhuay-Quispe J, Pacheco-Mendoza J (2019). Producción científica y licenciamiento de escuelas de medicina en el Perú. Rev Peru Med Exp Salud Publica.

[3] Dextre-Chacón JC, Tejedor S, Romero-Rodriguez LM (2021). Influence of institutional seniority and type of ownership on university quality rankings correlational analysis of Peruvian universities. J Appl Res High Educ.

[4] Paz-Enrique LE, Nunez-Jover JR, Hernandez-Alfonso EA (2022). Pensamiento latinoamericano en ciencia, tecnología e innovación políticas, determinantes y prácticas. Desde el Sur.

[5] Consejo Nacional de Ciencia.Tecnología e Innovación (2021). Reglamento de calificación, clasificación y registro de los investigadores del Sistema Nacional de Ciencia, Tecnología e Innovación Tecnológica - Reglamento RENACYT.

[6] Romaní FR, Wong P, Gutierrez C (2022). Formación de competencias en investigación científica basada en el diseño curricular en una facultad de medicina humana. An Fac Med (Lima).

[7] Mayta-Tristán P (2016). Tesis en formato de artículo científico oportunidad para incrementar la producción científica universitaria. Acta Med Peru.

[8] Quispe-Salcedo A (2021). Grupos de investigación juntos llegamos lejos. Rev Cient Odontol (Lima).

[9] Nieto-Gutierrez W, Fernandez-Chingel JE, Taype-Rondan A, Pacheco-Mendoza J, Mayta-Tristán P (2018). Incentivos por publicación científica en universidades peruanas que cuentan con escuelas de medicina, 2017. Rev Peru Med Exp Salud Publica.

[10] Ministerio de Educación (2022). DS 026-2022-EF: Establecen montos, criterios y condiciones de la bonificación especial para el docente investigador.

[11] Candal-Pedreira C, Ross JS, Ruano-Ravina A, Egilman DS, Fernandez E, Perez-Rios M (2022). Retracted papers originating from paper mills cross sectional study. BMJ.

[12] Day A (2022). Exploratory analysis of text duplication in peer-review reveals peer-review fraud and paper mills. Scientometrics.

[13] Santos-d'Amorim K, Wang T, Lund B, Dos Santos NR (2022). From plagiarism to scientific paper mills: a profile of retracted articles within the SciELO Brazil collection. Ethics Behav.

[14] Heck S, Bianchini F, Souren NY, Wilhelm C, Ohl Y, Plass C (2021). Fake data, paper mills, and their authors The International Journal of Cancer reacts to this threat to scientific integrity. Int J Cancer.

[15] Sotomayor-Beltrán C (2022). Awareness of predatory publishing ofr Peruvian university professors and lecturers doing research. Accountability in Research.

[16] Sotomayor-Beltrán C, Zarate GW (2022). Peruvian scientific production affected by predatory journals. Int Inf Libr Rev.

[17] Grundniewicz A, Moher F, Cobey K (2019). Predatory journals no definition, no defense. Nature.

[18] Besir S (2018). Predatory journals Who publishes in them and why?. J Infometrics.

[19] (2019). COPE council. Predatory publishing.

[20] Moreno-Loaiza O, Mamani-Quispe PV, Mayta-Tristán P (2013). Compra y venta de tesis online un problema ético por controlar. Rev Peru Med Exp Salud Publica.

[21] COPE & STM (2019). Paper Mills - Research report from COPE & STM.

[22] Perez-Neri I, Pineda C, Sandoval H (2022). Threats to scholarly research integrity arising from paper mills a rapid scoping review. Clin Rheumatol.

[23] Carnero AM, Mayta-Tristan P, Konda KA, Mezones-Holguin E, Bernabe-Ortiz A, Alvarado GF (2017). Plagiarism, cheating and research integrity case studies from a masters program in Peru. Sci Eng Ethics.

[24] Robishaw JD, DeMets DL, Wood SK, Boiselle PM, Hennekens CH (2020). Establishing and maintaining research integrity at academic institutions challenges and opportunities. Am J Med.

[25] Mejlgaard N, Bouter LM, Gaskell G, Kavouras P, Allum N, Bendtsen AK (2020). Research integrity nine ways to move from talk to walk. Nature.

