# Supplementary material for: Scientific misconducts: paper mills in Peru
Source: Rev Peru Med Exp Salud Publica. 2022 Dec 22;39(4):388–91. doi: 10.17843/rpmesp.2022.394.12473 (PMC11397752; doi:10.17843/rpmesp.2022.394.12473)
Supplement: Translation

# Translation: Malas prácticas en investigación: las fábricas de manuscritos en Perú

**Authors:** Percy Mayta-Tristán, Ruben Borja-García

**Affiliations:** 1 Dirección General de Investigación, Desarrollo e Innovación, Universidad Científica del Sur, Lima, Perú.

El Perú ha incrementado su producción científica en los últimos años catalizada por la reforma universitaria del 2014 y el proceso de licenciamiento de universidades y escuelas de medicina a cargo de la Superintendencia Nacional de Educación Superior (SUNEDU) ^1^ ^,^ ^2^. Además, las instituciones con mayor producción científica aparecen mejor posicionadas en los rankings de universidades ^3^ ^,^ ^4^; así mismo, sus docentes y estudiantes son reconocidos como investigadores (investigador RENACYT) por el Consejo Nacional de Ciencia, Tecnología e Innovación del Perú (CONCYTEC) ^5^.

Esta situación ha impulsado en la comunidad universitaria un mayor interés por la investigación y publicación de sus resultados en revistas indizadas, con cambios en las estrategias de formación en investigación como la aceptación de tesis en formato de artículo científico ^6^ ^,^ ^7^, desarrollo y apoyo a los grupos de investigación ^8^, también la generación de estímulos para los docentes que publican ^9^ incluido el bono de docente investigador que existe por ley para universidades públicas ^10^, entre otros. 

Una de las consecuencias de estos cambios es que, tanto investigadores como instituciones pueden privilegiar la publicación de ciertos tipos de manuscritos para aumentar el número de su producción, como las conferencias en congresos (*proceeding papers*) y cartas al editor (particularmente cartas comentario, del mismo tema, publicadas en diferentes revistas) que son de más rápida publicación, que se ven reflejados en una tendencia creciente en el país, especialmente los primeros (figura 2).

**Figura 2:** Evolución de la publicación de documentos de autores peruanos en Scopus según tipo documental, Scopus 2013-2021.

Las políticas de incentivo y reconocimiento de la investigación tienen un fin positivo, tanto para las instituciones, el país y los investigadores. Sin embargo, hay al menos cinco elementos en este proceso peruano que son un potencial caldo de cultivo para que ocurran malas prácticas en investigación si no hay los controles adecuados:

1. En el 2018 el CONCYTEC incluyó en los criterios de evaluación para investigadores RENACYT, además de bases de datos selectivas y reconocidas como Scopus, *Core Collection* de *Web of Science* (*Science Citation Index Expanded, Social Science Citation Index*) y SciELO, a una diversidad de editoriales y bases de datos (por ejemplo, Latindex) que no tenían los mismos filtros que las anteriores, así mismo incluyó en el puntaje la publicación de libros de investigación.
2. Un docente ordinario de una universidad pública que es reconocido como investigador RENACYT por CONCYTEC recibe un bono correspondiente al 50% de su sueldo de parte del Ministerio de Educación y una reducción de su tiempo de docencia a un curso por año.
3. Las universidades, principalmente privadas, brindan bonos por publicación de manuscritos en revistas indizadas a sus docentes e investigadores.
4. Para el nuevo proceso de licenciamiento institucional por SUNEDU, así como para el licenciamiento de medicina humana, se requiere acreditar un porcentaje de docentes que son RENACYT.
5. Una mayor preocupación de las universidades por aparecer y estar en una mejor posición en los rankings universitarios.

Esto ha contribuido a una cultura de «publicar o morir» que puede generar malas prácticas científicas como vicios de autoría (regalada, invitada) ^11^, publicación redundante ^12^, plagio ^13^, manipulación de datos ^14^, así como la publicación en revistas predadoras o carroñeras (*predatory journals*) ^15^.

Se ha descrito que al menos el 1% de la producción de las nueve universidades con mayor producción científica en el Perú se da en revistas potencialmente predadoras indizadas en Scopus, se ve una tendencia creciente con los años y se intuye que esto puede ocurrir más en otras universidades con un interés reciente por la investigación ^16^. Las revistas predadoras priorizan el interés propio y su único fin es el lucro. Se caracterizan por presentar contenido engañoso o falso, no siguen buenas prácticas editoriales y de publicación, carecen de transparencia y usan estrategias agresivas para solicitar la publicación de artículos ^17^. Además, suelen tener -si es que la tienen- una revisión por pares rápida e insustancial que no garantiza la calidad del contenido y periodos de publicación cortos ^18^ ^,^ ^19^.

En los últimos años, junto a la venta y compra de tesis ^20^, empezaron a proliferar anuncios de empresas locales dedicadas a «ayudar» a docentes universitarios a convertirse en investigadores RENACYT, ofreciéndoles, en el mejor escenario, publicar sus estudios o tesis en revistas incluidas en Latindex, muchas veces vinculadas a la misma empresa. En otros casos, ofrecen todo el proceso de investigación hasta la publicación, sin la certeza de que hubo un estudio real detrás (habría manipulación de datos o plagio). Se han detectado también anuncios de empresas que ofrecen y compran autorías, cuyo costo se incrementa según sea mayor el nivel de indización e impacto de la revista. 

Este tipo de organizaciones fraudulentas se denominan «fábricas de manuscritos» (*papers mill*) y son un problema de descripción reciente y en pleno crecimiento a nivel mundial, que tienen como fin distorsionar los procesos de investigación y publicación científica ^11^ ^-^ ^14^. El *Committee of Publication Ethics* (COPE) lo describe como «el proceso mediante el cual los manuscritos fabricados se envían a una revista a cambio de un costo en nombre de los investigadores con el fin de proporcionar una publicación fácil o incluso vender autorías» ^21^. Estas fábricas de manuscritos pueden incluir una o más de estas prácticas, que se agrupan en escritura fantasma de manuscritos, simulación del proceso de investigación, compra y venta de manuscritos y manipulación del proceso de investigación (tabla 2) ^22^.

**Tabla 2:** Tipificación de las prácticas de las fábricas de manuscritos. ^a^

Prácticas de las fábricas de manuscritos (artículos científicos)
Escritura fantasma de manuscritos: otra persona que no es parte de la lista de «autores» redacta el manuscrito a publicar. Puede tener diferentes modalidades:
De manuscritos «pseudo-originales» que incluyen datos que no son propios, estos pueden ser falsos, robados o manipulados; comprende también la manipulación de imágenes. Pueden ser revisiones sistemáticas con o sin metaanálisis, estudios bibliométricos o *proceeding papers.*
De artículos de revisión, comentarios, ensayos o cartas al editor.
Traducción de manuscritos publicados por primera vez en un idioma que no es el inglés y luego enviado a una revista en inglés, o viceversa (particularmente en caso de tesis, del inglés a español).
De tesis y trabajos de investigación académicos con o sin datos reales. Usan el término «asesoría» como fachada para la escritura fantasma. ^b^
Simulación del proceso de investigación, principalmente para tesis o trabajos institucionales. ^b^
Elaboración del protocolo de investigación y gestión del proceso de aprobación y sustentación. ^b^
Suministro de bases de datos plagiadas o fraudulentas.
Compra y venta de manuscritos.
Compra de artículos a investigadores, con el fin de ofrecer el artículo completo o posiciones de autoría.
Venta de autoría o posición en la autoría en manuscritos donde no han participado.
Manipulación del proceso de publicación.
Asegurar publicación rápida en revistas gestionadas por ellos o empresas vinculadas, sin proceso de revisión. En algunos casos estas revistas oficialmente no cobran por publicar para no ser consideradas predadoras. ^b^
Revisión por pares falsa o manipulación del proceso de revisión, incluye la posibilidad de suplantar revisores a través del envío de cuentas de correo falsas de verdaderos investigadores, cuando sugieren revisores para el artículo.
Servicios de selección y envío y seguimiento a revistas científicas, particularmente a revistas predadoras vinculadas o no con ellos. Esto puede incluir el uso de un correo falso del «autor corresponsal» o la gestión directa de una cuenta de correo que no es del «autor corresponsal».
Publicación de conferencias de congresos (*proceeding papers*) gestionados por ellos mismos. ^b^
Publicación de «libros de investigación» que puede ser una tesis o una investigación propia o simulada en el formato de un libro con ISBN gestionados por ellos. Puede incluir un certificado falso de revisión por pares. ^b^

a Adaptado de Perez-Neri *et al* ^22^.

b Agregado a partir de las prácticas evidenciadas en Perú.

Tipificar este tipo de mala práctica, que a su vez incluye a otras dentro de sí, es necesario para su detección y sanción. En primer lugar, se debe identificar cuáles son las fábricas de manuscritos que operan en el país y las revistas asociadas a ellas. Así mismo, a las personas que las promueven, quienes de ser investigadores afiliados a alguna institución académica deberían ser investigadas y sancionadas.

En algunos casos puede ser que el docente, por desconocimiento, adquiera este servicio pensando que es una forma de apoyo para la redacción y publicación de artículos, empero en otros hay intencionalidad directa de cometer fraude dado que buscan ser autores de investigaciones que no han hecho o que conociendo los procesos prefieren esta vía para llegar rápido a la meta de ser reconocido como investigador. Al igual que sucede con los casos de plagio ^23^, la tipificación de la falta «uso de fábricas de manuscritos» existe y la sanción dependerá de los factores agravantes y atenuantes identificados en el proceso de indagación.

Hoy, parte del problema se ha solucionado con el cambio de requisitos para ser investigador reconocido por CONCYTEC al ya no incluir a Latindex en la clasificación ^5^; sin embargo, quedan dos retos en relación con el control de la función editorial de estas fábricas de manuscritos. El primer aspecto es que algunas de estas empresas fraudulentas tienen revistas que se encuentran en Latindex y que, al ya no ser un indicador de evaluación por CONCYTEC, van a solicitar su inclusión en SciELO o en *Emerging Source Citation Index* de Web of Science, que podrían ser más accesibles que ingresar a Scopus o al *Science Citation Index Expanded*. En segundo lugar, algunas ofrecen la publicación de «libros de investigación» e inclusive congresos científicos donde garantizan la publicación de los trabajos en sus revistas o como libros, o capítulos que ellos también editan (tabla 2). El CONCYTEC a través de su Comité Nacional de Integridad Científica debe hacer un esfuerzo por identificar, investigar y sancionarlas cuando corresponda.

Para desincentivar el uso de fábricas de manuscritos las universidades deben capacitar a sus docentes y estudiantes sobre los procesos correctos de investigación y publicación, mas es necesario también identificar, investigar y sancionar a quienes usen estos servicios; para ello deben funcionar las Oficinas/Unidades/Comités de Integridad Científica que son diferentes de los Comités Institucionales de Ética en Investigación, puesto que a diferencia de estos -que buscan la protección del sujeto de investigación- los primeros velan por las buenas prácticas de investigación y tienen la facultad de investigar y sancionar a quienes cometen faltas ^24^ ^,^ ^25^. 

Finalmente, es necesario continuar con las políticas de impulso a la investigación ya implementadas, pues contribuyen al desarrollo del país. La integridad y la honestidad son características inherentes a la labor de investigación, tanto a nivel de individuos como de instituciones. Por ello, se debe identificar, investigar y sancionar las malas prácticas que pueden aparecer, poniendo énfasis en aquellas en crecimiento como las fábricas de manuscritos.
